# A retrospective cohort study on the association between mothers’ prolonged internet usage and severe early childhood caries in their children

**DOI:** 10.1186/s12887-025-05873-5

**Published:** 2025-07-02

**Authors:** Aya Sakakihara, Chiyori Haga, Aya Kinjo, Yoneatsu Osaki

**Affiliations:** 1 Department of Community Health Nursing, Faculty of Medicine, Shimane University, 89-1 Enya-cho, Izumo city, 693-8501 Shimane prefecture Japan; 2 Graduate School of Medicine, Department of Community Nursing, Kagawa University, 1750-1 Ikenobe, Miki-cho, Kita-gun, 761-0793 Kagawa Prefecture Japan; 3 Division of Environmental and Preventive Medicine, Faculty of Medicine, Tottori University, 86 Nishi-cho, Yonago-city, 683-8503 Tottori prefecture Japan

**Keywords:** Mothers’ prolonged internet usage, Problematic internet use, Childhood caries, ECC, Cohort study

## Abstract

**Objectives:**

Severe early childhood caries (S-ECC) is a child health challenge associated with neglect and represents a public health concern that can impact development and future lifestyle-related diseases. Despite its significance, studies identifying its associated factors have been scarce in developed countries. The aim of this study was to clarify the association between mothers’ prolonged Internet usage when their children are 1.5 years old and S-ECC at 3 years old.

**Methods:**

We included mothers who had notified Matsue City of their pregnancies during the 18-month period from April 2016 to September 2017, and their children. The data provided by the city were 2,465 records, with a follow-up rate of 82.5% from pregnancy notification to child health examination at Age 3. Excluding cases lacking information or indications of the presence of dental caries at Age 1.5, we analyzed 1,938 records. We performed logistic regression analysis, with S-ECC as the dependent variable and the mother’s Internet usage time at Age 1.5 as the independent variable, while including parental toothbrushing supervision and other factors as covariates.

**Results:**

The mother’s daily Internet usage time at Age 1.5 was more than 5 h in 2.0%. Children classified as having S-ECC at Age 3 accounted for 2.6%. We found a significant association between mothers’ Internet usage time at Age 1.5 and S-ECC in their children at Age 3 when the daily Internet usage time was 5 h or longer (adjusted odds ratio = 4.27 [95% CI: 1.42–12.86]).

**Conclusions:**

These findings suggest that Internet usage for 5 h or longer/day by mothers is associated with an increased likelihood of S-ECC in their children.

## Introduction

The number and accessibility of websites the Internet specifically targeting parents have increased [1]. Mothers may access a wide range of information using the Internet and receive emotional support [2–4]. However, the use of mobile devices by mothers during interactions with their children has been shown to disrupt parent-child interactions [5–7]. In the case of mothers who habitually spend a lot of time on the Internet, interactions with their children are difficult to restore [5]. Prolonged Internet usage is associated with problematic Internet usage (PIU), which has negative effects on interpersonal relationships, social life, and emotional stability [8, 9]. Previous studies reported that maternal PIU or mothers’ smartphone dependency was associated with mothers’ recognition of child abuse [10], rejection/neglect [11], thinness among their children during infancy [12], and problem behaviors among preschoolers, such as aggression, defiance, and emotional instability [13]. Due to their cross-sectional nature, it was difficult for these studies to prove causality; however, the findings obtained suggest that mothers’ inappropriate use of the Internet has a negative effect on parenting and children if it leads to decreased interest in children and a situation akin to neglect.

One of the child health challenges associated with neglect is early childhood caries (ECC), which often presents as multiple ECC lesions [14, 15]. ECC has a prevalence of 70% in developing countries and poor populations in developed countries [16], and is a public health concern [17]. Childhood caries is associated with being underweight and having stunted growth [18] as well as with obesity [19], and also leads to obesity and atherosclerosis in adulthood [20–22]. Severe ECC (S-ECC) may contribute to reduced sociability and self-esteem, with affected children becoming too embarrassed to laugh and unable to play with their friends [23]. Therefore, the prevention of ECC is important not only for oral health, but also for preventing lifestyle-related diseases and maintaining quality of life. In addition to parental neglect, factors associated with ECC include child characteristics, such as sex and birth weight; the parental work status; the parenting status, such as feeding habits and providing snacks to the child; and the family economic status [24–31]. However, the prevalence of ECC has not decreased in the past 25 years [17] despite the identification of many contributing factors, which emphasizes the importance of identifying other factors. Furthermore, studies to identify factors associated S-ECC have been conducted in some developing countries, but rarely in developed countries [32]; therefore, further research is urgently needed.

Oral health behaviors are the exclusive domain of parents during the early years of life [33]. We hypothesized that prolonged Internet use by mothers may encroach upon time spent on childcare, leading to the neglect of children’s oral care and, ultimately, the development of ECC. Childcare priorities for mothers typically include feeding, sleep, and toilet training [34, 35]. Among various health-related tasks, oral care is often considered to be a lower priority [36, 37]. Therefore, it is plausible that excessive Internet use may lead to the neglect of children’s oral care, thereby increasing the risk of ECC. Among previous studies, only one cross-sectional study examined the relationship between parental media use and ESS, and showed that parental media use for 2 h or longer was not associated with ECC [38]. Therefore, it is necessary to examine the relationship between Internet use longer than 2 h by parents and ECC in cohort studies. Caries may become clinically evident as early as 12 to 16 months after birth, for each year of age, children are likely to have ECC [39]. In the present study, we aimed to clarify whether mothers’ prolonged Internet usage when their children are 1.5 years old (Age 1.5) is associated with S-ECC when these children are 3 years old (Age 3). Identifying further parental lifestyle factors associated with ECC will contribute to increasing parental approaches to ECC prevention.

## Methods

### Study design and data sources

We conducted a retrospective cohort study in Matsue City, Shimane Prefecture. Matsue City is a provincial city with a population size of approximately 200,000 and 1,600 births/year. We enrolled mothers who had notified Matsue City of their pregnancies during the 1.5-year period from April 2016 to September 2017, and their children. In Japan, each municipality conducts health examinations for infants and Age 1.5 and Age 3 children to confirm their growth and development, and to address issues related to parenting in accordance with the Maternal and Child Health Act. In Matsue City, relevant children received child health examinations at Age 1.5 at a high rate of 99.6% (1,677/1683) in 2018 and 99.7% (1567/1571) in 2019, and child health examinations at Age 3 at 98.8% (1555/1574) in 2020 and 99.5% (1473/1481) in 2021. The city conducts a questionnaire survey on maternal Internet use at the times of pregnancy notification and infant health examinations. We used the results of the questionnaire survey and infant health examinations as secondary data for the present study. Three sets of data were combined: data on Internet usage filled in by mothers at the times of pregnancy notification and infant health examinations; basic information obtained at the time of birth notification, such as family structure, mother’s age, and child’s birth weight; and data obtained at the time of infant health examinations, including the child’s growth/developmental status, living condition, and parenting status. The data were provided to us with identification codes assigned. Names, addresses, district names, and birth dates as personally identifiable information were previously deleted. Before receiving the data, we posted an outline of the study on the websites of Shimane University and Matsue City, and provided an opportunity for the subjects to refuse the use of their data. This study was approved by the Medical Research Ethics Committee, Shimane University Faculty of Medicine (approval number: 5923).

**Fig. 1:**
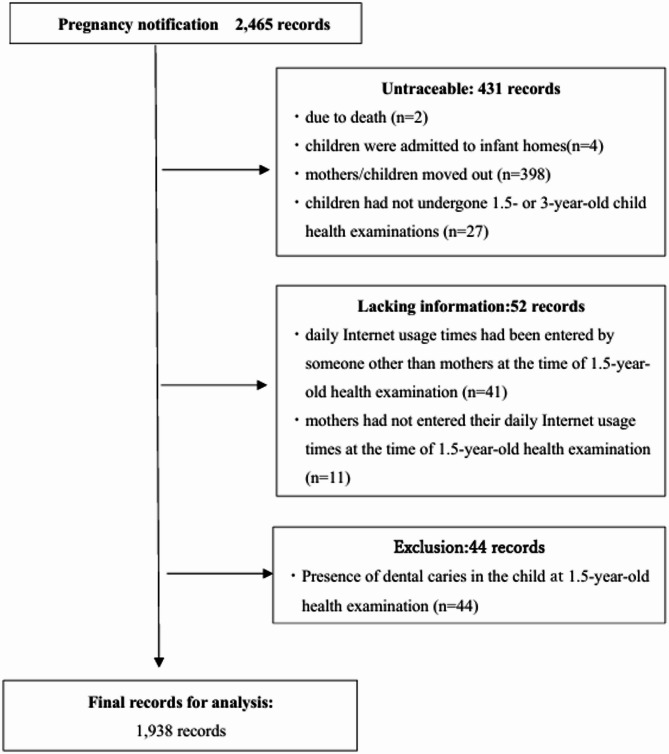
Flowchart of participant selection process

The data provided consisted of 2,465 records. Among these, there were two records in which children were untraceable due to death, four records in which children were admitted to infant homes, and 398 records in which mothers/children moved out. The follow-up rate from pregnancy notification to child health examinations at Age 3 was 82.5%. In addition, we excluded the following records: 27 in which children had not undergone child health examinations at Age 1.5 or Age 3; 41 in which daily Internet usage times had been entered by someone other than mothers at Age 1.5 child health examinations; 11 in which mothers had not entered their daily Internet usage times at Age 1.5 child health examinations; and 44 in which children had already had dental caries at the time of Age 1.5 child health examinations. We analyzed 1,938 records (Fig. 1).

### Measurements

#### 1) S-ECC

Based on the results of dental examinations for 3-year-old children by dentists and the definition of S-ECC for 3-year-olds by the American Academy on Pediatric Dentistry, we considered a decayed, missing, or filled number of ≥ 4 teeth as S-ECC in the present study [40].

#### 2) internet usage time and purpose of internet use

Internet use for ≥ 5 h/day is associated with PIU [41, 42]; therefore, persons who reported “≥5 hours” to the following question at the time of Age 1.5 child health examinations were evaluated as utilizing the Internet for many hours: “How many hours per day on average have you used the Internet in the past 30 days? Internet use indicates the use of a personal computer, cellular phone, smartphone, or tablet, and includes game or mail usage. It does not include Internet use for work.” The respondents selected their answers from the following options: “Never,” “Less than 1 hour,” “1–2 hours,” “2–3 hours,” “3–5 hours,” and “More than 5 hours.”

In addition, to clarify their purposes of Internet use, we asked the mothers to select from the following 8 options to answer another question presented at the time of Age 1.5 child health examinations, “Please select the purpose for which you have spent the most time using the Internet in the past 30 days”: (1) gathering information, (2) communicating with others, (3) disseminating information, (4) playing games, (5) shopping, (6) watching videos, (7) showing the child, and (8) others.

#### 3) covariates

Referring to the factors associated with ECC shown in previous studies [24–31] and those associated with mothers’ smartphone dependency, such as “being unemployed” [43] and “young age” [13], we considered the following variables as potential covariates: the child’s sex (male or female) and birth weight (≥ 2,500 or < 2,500), number of children (> 1 or 1), maternal age at birth (≥ 25 or < 25), daytime caregiver (family members including the mother or day-care centers), breast-/formula feeding (yes or no), number of snacks (< 3 or ≥ 3), parental toothbrushing supervision (yes or no), and parental smoking (yes or no) at Age 1.5 as covariates. While parental smoking is a factor associated with ESS [26], we included smoking as a proxy variable for socioeconomic status, based on the finding that smoking is more prevalent among individuals of low socioeconomic status [44]. Among these variables, only those that showed significant differences in the univariate analysis were included as covariates.

### Statistical analysis

We performed logistic regression analysis, with S-ECC at Age 3 as a dependent variable and the mother’s Internet usage time at Age 1.5 as an independent variable. We calculated the odds ratios and 95% confidence intervals for the mother’s Internet usage of more than 5 h, using less than 5 h as the reference. Among the covariates considered, we performed a multivariable logistic regression analysis using the variables that showed associations in the univariable logistic regression analysis. To assess the goodness of fit of the logistic regression model, we conducted the Hosmer-Lemeshow test.

Given that the mother’s Internet usage time may be double-counted in the case of multiple births, and that genetic factors may influence the risk of dental caries [45], we performed the same analysis, excluding multiple birth data, as a sensitivity analysis.

IBM SPSS Statistics 27 was used for the analysis, with the significance level set at < 5%.

## Results

The mother’s daily Internet usage time at Age 1.5 was more than 5 h in 2.0%. Children classified as having S-ECC at Age 3 accounted for 2.6%. Gathering information was the most common purpose (59.5%) for which mothers spent the most time using the Internet. However, among those who spent more than 5 h on the Internet, the rate of spending the most time on the Internet to gather information was 26.3%. The prevalence of dental caries among all children at Age 3 was 13.5%. (Table 1). Children classified as having S-ECC at Age 3 accounted for 2.6%. The prevalence of dental caries among all children at Age 3 was 13.5%. (Table 2)

**Table 1:** Characteristics of mothers

	*n*	%
Mother’s daily Internet usage time at Age 1.5 (*n* = 1,938)		
Never	5	0.3
Less than 1 h	683	35.2
1–2 h	777	40.1
2–3 h	309	15.9
3–5 h	126	6.5
≥ 5 h	38	2.0
The purpose for which the mother spent the most time using the Internet at Age 1.5 (*n* = 1,927)		
Gathering information	1153	59.5
Others than gathering information	774	39.9
Maternal Age (*n* = 1,938)		
≤ 25	159	8.2
> 25	1779	91.8
Parental toothbrushing supervision at Age 1.5 (*n* = 1930)		
No	92	4.7
Yes	1838	94.8
Parental smoking at Age 1.5 (*n* = 1,931)		
Yes	641	33.1
No	1290	66.6

**Table 2:** Characteristics of children

	*n*	%
Number of dental caries in the child at Age 3 (*n* = 1,938)		
None	1676	86.5
1–3	211	10.9
≥ 4	51	2.6
Birth weight (*n* = 1,938)		
< 2,500	180	9.3
≥ 2,500	1758	90.7
Number of children (*n* = 1,938)		
> 1	1147	59.2
1	791	40.8
Daytime caregiver at Age 1.5 (*n* = 1,936)		
Day-care centers	1555	80.2
Family members including the mother	381	19.7
Number of snacks at Age 1.5 (*n* = 1,929)		
≥ 3	307	15.8
< 3	1622	83.7

In univariate logistic regression analysis, the mother’s Internet usage at Age 1.5 was significantly associated with S-ECC at Age 3 when the daily Internet usage time was more than 5 h (odds ratio = 4.64 [95%CI: 1.58–13.60]) (Table 3). In the multivariable analysis, we included parental toothbrushing supervision at Age 1.5 and parental smoking at Age 1.5 as covariates, as they showed significant differences in the univariate logistic regression analysis. The multivariable analysis also demonstrated a significant association (adjusted odds ratio = 4.27 [95%CI: 1.42–12.86]) (Table 3).

**Table 3:** Odds ratios and 95% confidence intervals for the association between S-ECC at age 3 and mothers’ internet usage times at age 1.5

		Crude			Adjusted		
		OR	95%CI	P	OR	95%CI	P
	< 5 h	ref			ref		
Mother’s daily Internet usage time at Age 1.5	≥ 5 h	4.64	1.58–13.60	0.005	4.27	1.42–12.86	0.010
Parental toothbrushing supervision at Age 1.5	Yes	ref			ref		
	No	2.84	1.18–6.86	0.020	2.70	1.10–6.61	0.030
Parental smoking at Age 1.5	no	ref			ref		
	Yes	3.51	1.98–6.25	< 0.001	3.62	2.01–6.52	< 0.001

OR: odds ratio; CI: confidence interval; ref: reference

Adjusted for parental toothbrushing supervision at Age 1.5 and parental smoking at Age 1.5

The results of the sensitivity analysis, excluding multiple birth data, remained consistent.

There was no multicollinearity of the entered variables. The goodness of fit of the model based on the Hosmer-Lemeshow test was *p* ≥ 0.05.

## Discussion

This study examined the association between mothers’ prolonged Internet usage when their children are 1.5 years old and S-ECC at 3 years old. The results suggested that children of mothers who used the Internet for 5 h or longer/day when the children were 1.5 years old are 4.24 times more likely to have S-ECC at the age of 3, compared with children of mothers who did not.

Since the association between prolonged Internet use by mothers and S-ECC in children remained significant even after adjusting for parental-assisted toothbrushing at 1.5 years of age, it cannot be concluded that mothers who use the Internet for extended periods are not assisting with their children’s toothbrushing. However, Previous experiments on technoference reported that when mothers use mobile devices, even temporarily, they may become less responsive to their children and distracted during use [5, 46]. A case report on maternal PIU reported that these mothers were distracted from housework and parenting [47]. Furthermore, an increased risk of neglect due to immersion in the Internet has been suggested among mothers with PIU or smartphone dependency [10, 11]. Mindfulness [48], which is the ability to focus on the immediate task, also decreased. Therefore, mothers who are habitually engrossed in the Internet may pay insufficient attention to brushing their children’s teeth, being unable to do so carefully and thoroughly, as part of parental toothbrushing supervision, and they may even be unaware of the presence of dental caries in their children.

Additionally, previous studies demonstrated that PIU was associated with lower toothbrushing frequency [49–51], and parental toothbrushing habits were found to be associated with the frequency of parental-assisted toothbrushing [52]. These findings suggest that mothers who engage in prolonged Internet use perform parental-assisted toothbrushing less frequently.

In other words, it is possible that mothers who use the Internet for extended periods may not perform parental-assisted toothbrushing carefully or frequently, which could be one of the mechanisms linking prolonged Internet use to S-ECC. Further research is needed to clarify the mechanisms underlying the relationship between prolonged maternal Internet use and S-ECC by incorporating additional data, such as the frequency of children’s dental check-ups and sugar intake.

Based on a previous study [53] showing that mothers spent an average of approximately 3 h per day on the Internet, spending more than 5 h per day on the Internet may result in cutting back on parenting duties. In the present study, 2.0% of mothers had used the Internet for more than 5 h daily when their children were 1.5 years old. Since the prevalence of PIU among mothers was reported to be approximately 1–3% [12], a daily Internet usage time of more than 5 h may be excessive and problematic.

Mothers typically prioritize childcare tasks such as feeding, sleep, and toilet training [34, 35]; therefore, the aspects of childcare that may be omitted due to prolonged Internet use are likely to be related to children’s oral health care, such as parental toothbrushing supervision, dental visits, and fluoride application. Therefore, it is important to utilize opportunities, such as routine health check-ups for infants and young children, to assess whether prolonged Internet use by mothers may be compromising their caregiving—particularly their children’s oral health—and to provide guidance on the importance of prioritizing direct child care over Internet use.

The gathering of information using the Internet is beneficial for parenting [2–4]; however, among mothers whose Internet usage exceeded five hours per day, it was not a common primary purpose for which they spent most of their Internet time. Encouraging mothers to use the Internet in order to access parenting-related information, while minimizing time spent on activities such as gaming or video streaming may be a more effective approach.

The present study has the following limitations. First, as the data were collected from a single local city, they are not representative of Japan, and there may have been a sampling bias. The small number of S-ECC cases and the limited number of mothers with prolonged Internet use may have resulted in insufficient statistical power and instability in the estimates. In future studies, it will be necessary to increase the sample size by including data from multiple municipalities, including participants from urban areas. Second, mothers’ daily Internet usage times were based on their self-reports, which may have introduced an information bias. Mothers may not have been able to accurately recall their Internet usage time over the past 30 days, which could have led to underreporting or overreporting [54]. In future studies, it will be necessary to use objective measures of Internet usage (e.g., screen time tracking apps [55, 56]) for analysis. Third, a major confounding factor for dental caries may be the socioeconomic status, such as the maternal education level, income, and employment status [24, 25, 29–31]; however, it was not fully adjusted for by the use of parental smoking as a covariate in this study. Future follow-up studies using variables that accurately represent the socioeconomic status, such as maternal education and household income, are required. Fourth, the study only examined mothers, but fathers are also important in parenting [57]. Thus, it is necessary to include fathers in future studies to investigate the relationship between parents’ prolonged Internet usage and their children’s growth and development. Fifth, since this was a retrospective cohort study, causal relationships cannot be established. Therefore, future studies using a prospective cohort design are needed.

Despite these limitations, the present study is of significance in identifying mothers’ prolonged Internet usage as a new factor associated with S-ECC. Future studies should accumulate evidence on the relationship between parents’ prolonged Internet usage and children’s growth and development, adopting study designs that overcome these limitations.

## Conclusions

These findings suggest that Internet usage for 5 h or longer/day by mothers is associated with an increased likelihood of S-ECC in their children.

## Data Availability

The datasets analysed during the current study are not publicly available as they are provided and owned by Matsue City, Shimane Prefecture; however, they are available from the corresponding author on reasonable request.

## Abbreviations

PIU: Problematic Internet use
S-ECC: Severe early childhood caries
